# Supplementary material for: Connectome-based reservoir computing with the conn2res toolbox
Source: Nat Commun. 2024 Jan 22;15:656. doi: 10.1038/s41467-024-44900-4 (PMC10803782; doi:10.1038/s41467-024-44900-4)
Supplement: Supplementary file 1 — Supplementary Information [file 41467_2024_44900_MOESM1_ESM.pdf]

Connectome-based reservoir computing with the conn2res toolbox

package name	network architecture	local dynamics	task paradigms	learning algorithm	performance metrics
conn2res	Arbitrary	Artificial neuron models with different types of nonlinearities, spiking neurons (leaky-integrate-and-fire neurons) and memristive dynamics for neuromorphic devices (metastable switch model).	Includes classic RC tasks such as memory capacity, all behavioural paradigms included in the NeuroGym repository (e.g., context decision making, delay match category, delay match sample, interval discrimination, plus 15+ more), as well as all the non-neuroscience-related tasks included in the ReservoirPy package (e.g., prediction of chaotic time series such as Hénon map or Mackey-Glass time series) are also available.	All linear models present in the Scikit-learn package are available.	Regression metrics: r-squared / coefficient of determination (R^2), mean square error (MSE), root mean squared error (RMSE), normalized root mean squared error (NRMSE), mean absolute error (MAE), correlation coefficient. Classification metrics: accuracy, balanced accuracy, f1-score, precision and recall.
ReservoirPy	Arbitrary	Leaky integrator neurons.	Tasks included are not necessarily neuroscience related (e.g., prediction of chaotic time series such as Hénon map or Mackey-Glass time series).	Logistic regression, Ridge classification and perceptron. Others include Recursive Least Squares and the Least Mean Squares learning rule.	Regression metrics: mean square error (MSE), root mean squared error (RMSE), normalized root mean squared error (NRMSE), r-squared / coefficient of determination (R^2).
echoes	Arbitrary	Leaky integrator neurons.	Prediction of chaotic Mackey-Glass time series.	Ridge regression.	Regression metrics: r-squared / coefficient of determination (R^2).

TABLE S1. The conn2res toolbox - comparison with other RC-based Python packages.
